# Alleged Detrimental Mutations in the *SMPD1* Gene in Patients with Niemann-Pick Disease

**DOI:** 10.3390/ijms160613649

**Published:** 2015-06-15

**Authors:** Cosima Rhein, Christiane Mühle, Johannes Kornhuber, Martin Reichel

**Affiliations:** Department of Psychiatry and Psychotherapy, Friedrich-Alexander-University Erlangen-Nuremberg, Schwabachanlage 6, 91054 Erlangen, Germany; E-Mails: cosima.rhein@uk-erlangen.de (C.R.); christiane.muehle@uk-erlangen.de (C.M.); johannes.kornhuber@uk-erlangen.de (J.K.)

**Keywords:** gene variant, missense mutation, Niemann-Pick disease, polymorphism, sphingomyelin phosphodiesterase

## Abstract

Loss-of-function mutations in the sphingomyelin phosphodiesterase 1 (*SMPD1*) gene are associated with decreased catalytic activity of acid sphingomyelinase (ASM) and are the cause of the autosomal recessive lysosomal storage disorder Niemann-Pick disease (NPD) types A and B. Currently, >100 missense mutations in *SMPD1* are listed in the Human Gene Mutation Database. However, not every sequence variation in *SMPD1* is detrimental and gives rise to NPD. We have analysed several alleged *SMPD1* missense mutations mentioned in a recent publication and found them to be common variants of *SMPD1* that give rise to normal *in vivo* and *in vitro* ASM activity. (Comment on Manshadi *et al*. *Int. J. Mol. Sci.* **2015**, *16*, 6668–6676).

## 1. To the Editor

Loss-of-function mutations in the sphingomyelin phosphodiesterase 1 (*SMPD1*) gene are associated with decreased catalytic activity of acid sphingomyelinase (ASM) and are the cause of the autosomal recessive lysosomal storage disorder Niemann-Pick disease types A and B (NPD). Currently, >100 missense mutations in *SMPD1* are listed in the Human Gene Mutation Database. However, not every sequence variation in *SMPD1* is detrimental and gives rise to NPD.

**Figure 1:**
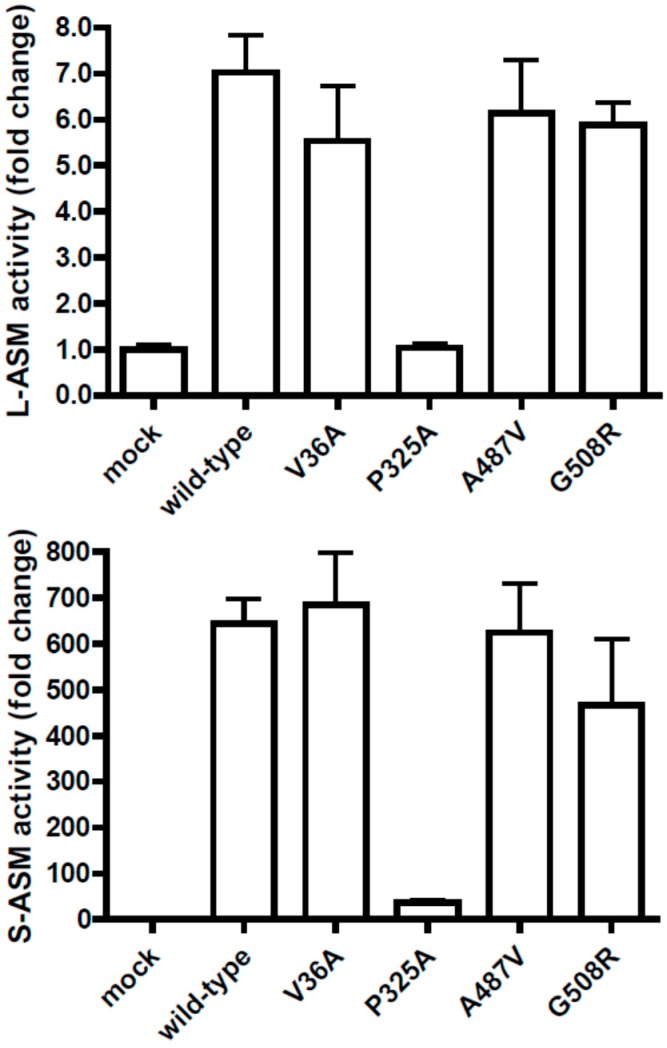
Analysis of sphingomyelin phosphodiesterase 1 (*SMPD1*) sequence variations by transient transfection studies. The full-length *SMPD1* cDNA was cloned into the FLAG-N2 expression vector, and the *SMPD1* variants p.V36A, p.P325A, p.A487V and p.G508R were generated by site-directed mutagenesis. The variant cDNAs and the empty FLAG-N2 vector (mock) were transiently transfected into MDCK cells, and acid sphingomyelinase activity was determined from cell lysates (L-ASM activity; **upper** panel) and supernatants (S-ASM activity; **lower** panel). Representative results of a typical experiment with three replicates are given as fold increase over the mock-transfected control. Error bars indicate the standard deviation. With the exception of p.P325A, all mutants increased acid sphingomyelinase activity by >fivefold, similarly to *SMPD1* wild-type. Methods are described in detail in [5].

In order to identify mutations in the *SMPD1* gene that potentially explain increased ASM activity associated with major depressive disorder [1] we performed a re-sequencing analysis of the six exons of *SMPD1*. This region includes a 1896 bp open reading frame that encodes a 631 amino acid protein according to NCBI Reference Sequence NM_000543.4. We identified three non-synonymous single nucleotide polymorphisms (SNP)-c.973C>G (p.P325A), c.1460C>T (p.A487V) and c.1522G>A (p.G508R; rs1050239)-as well as a bipartite polymorphic site including the non-synonymous SNP c.107T>C (p.V36A; rs1050228), which is located adjacent to a polymorphic region composed of a variable number of hexanucleotide sequences. Two of these variants, p.P325A and p.A487V, were previously reported to constitute loss-of-function mutations of *SMPD1* [2], whereas the other two, p.G508R and the hexanucleotide repeat, an association with NPD was ruled out [3,4]. To analyze the functional implications of these polymorphisms in detail, we first conducted genotype-phenotype correlations in different human samples (e.g., a comparison of individual *SMPD1* genotypes with the respective *in vivo* ASM activities) and, second, cloned the respective cDNAs for transient transfection studies. Hereby, we were able to demonstrate that the carriers of c.1460C>T (minor allele frequency ~1%) displayed secreted (S-) and lysosomal (L-) ASM activities in the normal range [5]. Moreover, transient transfection of c.1460T-cDNA into HeLa and MDCK cells resulted in S- and L-ASM activities similar to wild-type ASM [5]. The genotype-phenotype comparison for c.1522G>A (minor allele frequency ~27%), on the other hand, revealed a significant association especially with the S-ASM activity, which decreased with the number of A alleles in a gene-dosage dependent manner [6]. S-ASM activity in subjects homozygous for c.1522G>A was 50% decreased compared to subjects homozygous for the major allele c.1522G [6]. In sharp contrast, NPD patients and carriers of NPD alleles were reported to display S-ASM activities <3% and about 20% of normal values, respectively [7]. Thus, the only moderately decreased S-ASM activity in subjects homozygous for c.1522A and the high allele frequency argue strongly against the notion that c.1522G>A constitutes a loss-of-function mutation causing NPD. In addition, a transient transfection of a c.1522A-construct into HeLa and MCDK cells resulted in L- and S-ASM activities comparable to wild-type ASM (Figure 1). The functional relevance of c.107T>C was analyzed together with that of the adjacent polymorphic hexanucleotide repeat. While we obtained data showing that less repeats of the hexanucleotide motif are associated with slightly decreased S-ASM activity *in vivo* and *in vitro* [8], we did not observe a significant reduction of ASM activity associated with c.107T>C, neither *in vivo* nor *in vitro* (Figure 1). In contrast, transient transfection of a c.973G-construct into HeLa and MDCK cells did not increase ASM activity compared to control cells, thus confirming that c.973C>G is a NPD mutation (Figure 1).

## 2. Conclusions

Our results strongly support the notion that p.V36A, p.A487V and p.G508R are frequent polymorphisms in *SMPD1*. These polymorphisms might increase the susceptibility for common diseases such as allergy [6], but they do not constitute loss-of-function mutations that are responsible for the occurrence of NPD. Since the alleged loss-of function mutations in 12 out of 15 NPD patients reported by Manshadi *et al.* [9] are common polymorphisms, a critical molecular, biochemical and clinical evaluation of the mentioned patients is recommended.
